# The Alice Springs Hospital Readmission Prevention Project (ASHRAPP): a randomised control trial

**DOI:** 10.1186/s12913-017-2077-7

**Published:** 2017-02-20

**Authors:** Gabrielle Diplock, James Ward, Simon Stewart, Paul Scuffham, Penny Stewart, Carole Reeve, Lea Davidson, Graeme Maguire

**Affiliations:** 1 Monash University and Baker IDI Heart & Diabetes Institute, Melbourne, Australia; 2 South Australian Health & Medical Research Institute, Adelaide, Australia; 3 Menzies Health Institute Queensland, Griffith University, Brisbane, Australia; 4 Alice Springs Hospital, Alice Springs, Australia

**Keywords:** Readmission prevention, Transitional care, Discharge planning, Indigenous health, Health service intervention

## Abstract

**Background:**

Hospitals are frequently faced with high levels of emergency department presentations and demand for inpatient care. An important contributing factor is the subset of patients with complex chronic diseases who have frequent and preventable exacerbations of their chronic diseases. Evidence suggests that some of these hospital readmissions can be prevented with appropriate transitional care. Whilst there is a growing body of evidence for transitional care processes in urban, non-indigenous settings, there is a paucity of information regarding rural and remote settings and, specifically, the indigenous context.

**Methods:**

This randomised control trial compares a tailored, multidimensional transitional care package to usual care. The objective is to evaluate the efficacy of the transitional care package for Indigenous and non-Indigenous Australian patients with chronic diseases at risk of recurrent readmission with the aim of reducing readmission rates and improving transition to primary care in a remote setting. Patients will be recruited from medical and surgical admissions to Alice Springs Hospital and will be followed for 12 months. The primary outcome measure will be number of admissions to hospital with secondary outcomes including number of emergency department presentations, number of ICU admissions, days alive and out of hospital, time to primary care review post discharge and cost-effectiveness.

**Discussion:**

Successful transition from hospital to home is important for patients with complex chronic diseases. Evidence suggests that a coordinated transitional care plan can result in a reduction in length of hospital stay and readmission rates for adults with complex medical needs. This will be the first study to evaluate a tailored multidimensional transitional care intervention to prevent readmission in Indigenous and non-Indigenous Australian residents of remote Australia who are frequently admitted to hospital. If demonstrated to be effective it will have implications for the care and management of Indigenous Australians throughout regional and remote Australia and in other remote, culturally and linguistically diverse populations and settings.

**Trial registration:**

Australian New Zealand Clinical Trials Registry, ACTRN12615000808549- Retrospectively registered on 4/8/15.

**Electronic supplementary material:**

The online version of this article (doi:10.1186/s12913-017-2077-7) contains supplementary material, which is available to authorized users.

## Background

### The impact of frequent hospital attendance on patients and hospitals

Hospitals are frequently faced with high levels of emergency department presentations and demand for inpatient care. These demands often exceed the available resources especially in regional and remote areas where alternate hospitals or services are distant and difficult to access. Delayed emergency department review, admitted patients waiting in emergency departments and long hospital elective waiting lists can, in part, be traced back to a lack of inpatient beds [1–4]. An important contributing factor to this mismatch between the demand for inpatient care and its availability is the admission of an often small but important sub-set of patients who have frequent and preventable exacerbations of their chronic diseases [5]. This is often compounded by a lack of, or limited access to, community-based social and primary health care supports. Such hospital admissions remove an individual from their family and community supports, limit their ability to undertake usual activities and expose them to risk of infection and other nosocomial complications. Many of these are patients with chronic and other diseases that can, with appropriate community-based management and support, avoid de-compensation and the need for inpatient care [6–8].

Recurrent readmissions also pose a financial burden on a health system that is faced with escalating costs. In the US, nearly half of the total health care budget is spent on inpatient services. Existing studies have demonstrated that readmissions account for one quarter of total inpatient expenditure [9, 10]. Given its impact on total health care expenditure cost containment associated with acute inpatient care is an important focus [11]. This has encouraged the development of initiatives that aim to decrease length of inpatient stay, increase the use of day procedures, transfer care to the community setting including “hospital in the home” programs and reduce preventable hospital readmission [10, 12]. Evidence indicates that even a small reduction in readmission numbers could have a substantial financial benefit.

### Preventing repeated attendance and readmission to hospital

Existing literature suggests many hospital readmissions are related to preventable or avoidable causes [13]. Targets for initiatives that might reduce readmission include improved patient and family education and community-based support, pre-discharge planning and community and primary health care based liaison, early follow-up and ongoing chronic disease management. Despite many health care services allocating significant resources to facilitating hospital discharge and preventing readmission evidence supporting a particular approach is limited and largely lacking for disadvantaged, remote and Indigenous Australian populations.

One hospital-based readmission prevention initiative is discharge planning. This involves the development of an individualised plan for a patient prior to leaving hospital. The aim is to reduce length of hospital stay and unplanned readmission, and improve the coordination of hospital and community-based services following discharge [14]. Evidence suggests that a coordinated discharge plan tailored to the individual patient brings about a reduction in length of hospital stay and readmission rates for adults with complex medical needs. Specific interventions include formal assessment for risk factors relating to delayed and failed discharge, patient education, medication reconciliation, discharge care plans and post discharge follow-up including telephonic review, home visits and timely review with a primary care provider. Dedicated discharge planning staff to coordinate this process has been highlighted as an important contributor to the success of such programs [15, 16].

Many hospitals are already making a significant investment in attempting to reduce inpatient stays and readmission in the form of designated discharge planning positions and allied health, pharmacy, drug and alcohol, social work, mental health, aged, rehabilitation and palliative care services. While such services are variably available and accessed by all Australians, the need is even greater for remote residents, who already face limited access to health care, and Indigenous Australian patients who are more likely to face issues relating to a greater burden of co-morbidity, language, intercultural communication, remoteness and economic and environmental disadvantage.

### Alice Springs Hospital and frequent hospital admissions

Alice Springs Hospital is the regional referral centre for Central Australia. It has a catchment of approximately 50,000 residents incorporating Alice Springs and up to 50 remote communities that range between 80 and 1000 kms from the hospital. Indigenous Australians, who are often faced with significant and complex health needs, represent 40% of this population. A combination of a remote population with a high burden of complex and chronic disease can result in high levels of emergency patient presentations to Alice Springs Hospital and delays in transfer to inpatient wards which can in turn rapidly lead to an overcrowded emergency department, increased waiting times and, by extension, poorer patient outcomes [3]. As previously noted, an important potential factor contributing to this issue are patients who repeatedly attend hospital and require admission due to a combination of preventable exacerbations of their chronic disease and a lack of community-based support.

A particular group at risk of re-admission to Alice Springs Hospital are adult patients with, usually multiple, chronic but not immediately life threatening diseases such as chronic lung (Chronic Obstructive Pulmonary Disease (COPD)/bronchiectasis/asthma) and heart (heart failure, coronary artery disease) disease [17, 18]. In Alice Springs, many of these patients are Aboriginal people who may live in Alice Springs or remote communities up to 1000km distant from the hospital. In 2012, excluding oncology and renal dialysis patients, there were more than 320 adult medical patients (90% Aboriginal Australian) admitted to Alice Springs Hospital who had more than five admissions over the preceding year. These patients had an average of eight admissions per year and accounted for over 2500 admissions or approximately 25% of adult medical admissions. Thus, 320 adult medical patients or less than 1% of the adult population of Central Australia, accounted for one quarter of the admissions to the adult medical service at Alice Springs Hospital.

There is currently no consistent definition for unplanned hospital readmissions. This makes comparison of data difficult and as a result readmission data is not part of current hospital performance frameworks. Australian data indicate that across all hospitals (public and private) and age groups, 1% of patients are readmitted within 28-days [19]. A recent study at Flinders Medical Centre in South Australia found that just over 10% of patients were being readmitted within 28 days of discharge. Those patients with respiratory, neurological or genitourinary and trauma were more likely to be readmitted. Risk factors for readmission included increased length of stay, high co-morbidity index scores and discharge against medical advice [19].

Much of the current research relating to miminising hospital stay and readmission has been conducted with specific medical populations including heart failure or COPD [20–25]. Recent data from three randomised trials indicated that multidisciplinary home-based case management for patients hospitalised with the full spectrum of chronic heart disease are effective in prolonging days alive and out of hospital and avoiding recurrent hospitalisation [26]. Whilst some research has been conducted in vulnerable populations, including low socioeconomic or ethnic minority groups [27–29], such studies do not specifically address the unique aspects of discharge planning when dealing with vulnerable, remote populations with a high burden of disease. There are few specific Australian studies especially those dealing with a rural/remote context.

Given the lack of evidence, we aim to extend and adapt the principles of what are often disease-specific, non-Indigenous and urban based models of readmission prevention and to evaluate such a more generic model linking hospitals with primary health care and other community-based resources in a remote setting with a large Aboriginal Australian population. To achieve this we aim to evaluate the efficacy of a tailored multi-dimensional case-based intervention for people with frequent admissions to the adult specialist medical and surgical service of a remote Australian hospital with a particular focus on the needs of Aboriginal people.

## Hypothesis

Relative to standard management, a targeted case management and discharge planning process aimed at adult medical and surgical inpatients with a history of a) four or more admissions over the last 12 months or b) more than seven admissions over the previous 24 months will be effective in reducing the rate of all-cause rehospitalisation (primary endpoint) and health care utilisation in the 12 months following the index admission.

## Methods/Design

### Objectives

The primary objective of this study is to compare a tailored discharge planning and case management approach for patients who are frequently admitted to Alice Springs Hospital with patients receiving usual care using the primary outcome of decreased readmissions. To do this we will:

i. Develop and evaluate a multidimensional discharge planning and case-management approach aimed at reducing all-cause readmissions
ii. Identify factors which predict readmission and subsequent mortality and thus factors which identify patients who are most likely to benefit from this intervention
iii. Evaluate the efficacy and cost-effectiveness of this tailored case management-based approach

### Study design

**Fig. 1:**
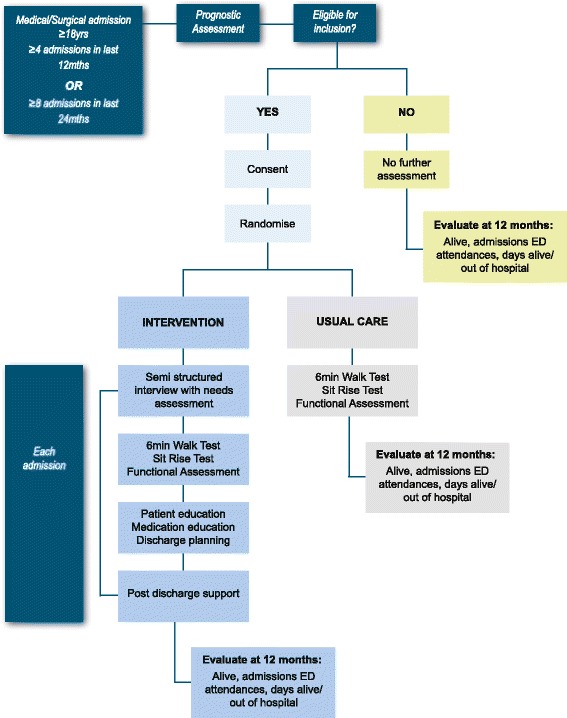
Patient flow through study protocol

In this randomised control trial conducted according to CONSORT guidelines for a pragmatic trial of a health service intervention, participants will be randomly allocated to either of two study arms using a concealed allocation procedure [30]. The study arms will include an intervention group who will participate in the case management approach over 12 months and a control group who will receive usual hospital-based care including discharge planning using the existing hospital-based, nurse led service. The case management approach will be delivered by a team consisting of a medical officer, Aboriginal Health Practitioner, nurse and pharmacist. Figure 1 summarises a patient’s journey through the study protocol.

Data collection will occur at enrolment, at each subsequent admission to hospital and at the conclusion of the study when primary health care utilisation and survival data will be collected. The primary outcome of interest will be a reduction in the number of admissions to hospital over the 12 month period following enrolment and will be based on intention to treat.

### Study setting

**Fig. 2:**
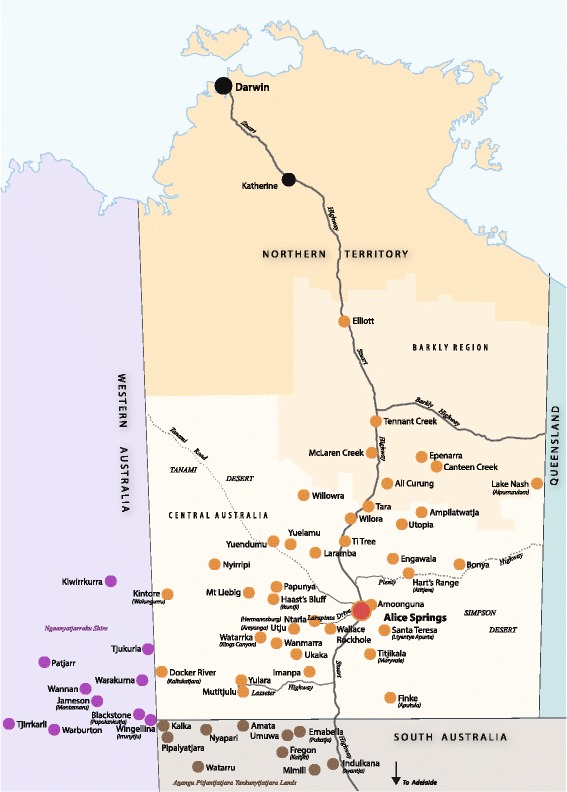
Map of catchment area for Alice Springs Hospital

This study will be conducted at Alice Springs Hospital, the regional referral centre for remote Central Australia with 186 inpatient beds. It is the major teaching hospital servicing approximately 50,000 residents of both Alice Springs and up to 50 remote communities that range between 80 and 1000kms from the hospital (See Fig. 2). The nearest tertiary hospital referral centres, Darwin and Adelaide, are approximately 1500 km away by road.

### Study population

Subjects will be identified within 48 h of admission under an adult, general medical or surgical team. Alice Springs Hospital does not have sub-specialty adult medical units (e.g. cardiology, gastroenterology etc.) but each of the three adult general medical units tend to focus on one or more medical sub-specialties. Surgical patients will be required to have one or more pre-existing chronic disease diagnoses which have contributed to the current admission (e.g. foot ulcer with diabetes). Whilst enrolment will not be restricted to Indigenous Australians the majority of people requiring frequent admission to Alice Springs Hospital are Aboriginal Australians.

### Inclusion criteria

1. 18 years and older
2. 4 or more adult medical and/or non-elective surgical admissions over the preceding 12 months or 8 over the preceding 24 months
  - Surgical admissions will be counted if deemed a consequence of a chronic disease such as diabetic foot infections, acute/chronic pancreatitis and cellulitis
3. Resident of Central Australia (including the cross border regions of Western Australia and South Australia and extending north to Elliot in the Northern Territory).

### Exclusion criteria

1. Anticipated life expectancy of 12 months or less based on treating specialist assessment
2. Stage 5 chronic kidney disease (eGFR < 15ml/min or receiving renal replacement therapy)
3. Solid organ transplant (including renal transplant)
4. Active palliative care involvement
5. Previously been enrolled in the study (such subjects will have on-going care as per their original study allocation)

### Randomisation: allocation, concealment and sequence generation

A computerised randomisation database using Microsoft Access will be utilised. Given the resource implications of the intervention, the randomisation ratio will be unequal with one participant allocated to the intervention group for every two allocated to the control group. Block randomisation will be used to randomly assign participants to intervention or usual care with participants being randomised in blocks of 21. Each block will have seven participants randomly assigned to the intervention and 14 participants to the control arm. Once eligibility is confirmed and the participant consented, the local study team will enter the enrolled participant’s details into the database and allocation will be automatically assigned.

Whilst allocation will be concealed the nature of the intervention will mean participants, research team member and local health care staff are aware of which group each participant has been allocated to. Staff assessing primary endpoint data and those involved with the analysis will be blinded to the study allocation.

### Intervention

Participants will be informed that they will be randomly assigned to a group that receives ‘usual care’ or a group that receives extra support in the form of a transitional care package.

#### Participants assigned to the control group

Participants allocated to the control group will receive usual care in the form of existing discharge planning services provided by Alice Springs Hospital. This includes a varying combination of patient education regarding their health conditions and referral to allied health services (e.g. physiotherapy, occupational therapy, dietician, speech pathology, social work, substance abuse and addiction, and pharmacy services).

#### Participants assigned to intervention group

**Fig. 3:**
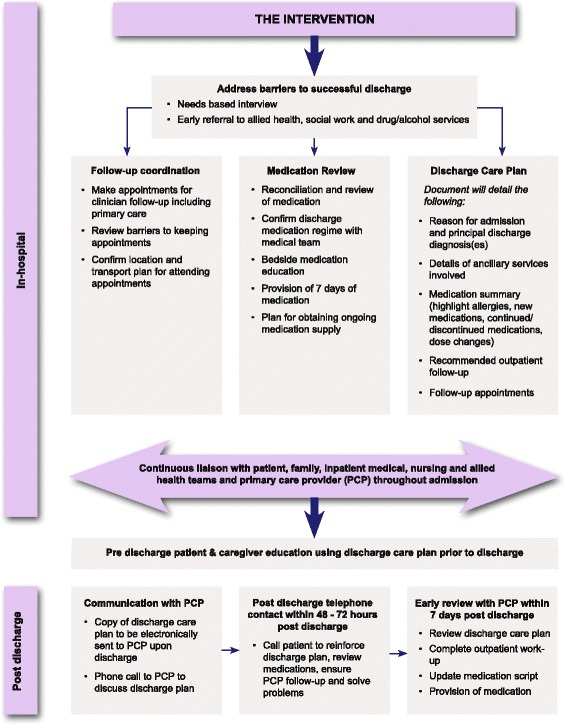
The intervention process

Participants allocated to the intervention group will receive the usual services as described above. In addition they will be provided with a multi-dimensional and case-based transitional care package led by a designated team consisting of a medical officer, nurse, Aboriginal Health Practitioner and pharmacist. At each admission, the participants will have the following provided during their inpatient stay. This is also summarised in Fig. 3:

1. A comprehensive needs based semi-structured interview (including health, social and other potential drivers of readmission)
2. Coordination of referrals to allied health, social work, mental health and/or substance abuse and addiction services based on this needs based assessment
3. Nurse and medical officer-led education to participant and family regarding diagnosis and principles of management supported by Aboriginal language interpreters and Aboriginal Health Practitioners
4. Full medication review, reconciliation and bedside education by the dedicated team pharmacist
5. Case conferencing with ward-based medical and nursing staff to develop a clear ongoing management plan including expectations regarding post-hospital management
6. Liaison with local primary healthcare providers (including negotiation regarding follow-up and ensuring high quality, informative discharge summaries are completed in a timely manner)
7. Development of a written discharge plan with the treating medical team, participant, primary health care provider and family with a copy being given to the participant and sent to a designated individual at the primary health care site (preferably by email) at the time of discharge

The intervention team will facilitate the following activities following discharge through liaison with the participant, family and/or primary health care provider:

1. Telephone case conference with patient and family between day 3 and 5 of discharge. Telephone contact was chosen over home visits given the potential remoteness of our patient population and the inherent difficulties in providing a home based service post discharge. This semi structured interview with focus on how the patient is coping post discharge, any complications/new problems, medication supply, adherence and side effects, a reminder of upcoming reviews and any potential barriers to attending these appointments. The researcher will provide advice and modify the management plan depending on outcomes of this phone interview.
2. Telephone case conference with primary care provider between day 1 and 5 post discharge. The researcher will provide the primary care provider with a summary of the admission and discharge plan and an opportunity to clarify any confusion. The researcher will also check that the primary care provider received the written discharge plan and that the patient has a review appointment booked.
3. Participant primary health care review within 7 days following hospital discharge.
4. Support for participants if they return to hospital for outpatient review and/or investigations to encourage ambulatory service attendance and to consolidate understanding of management plans and expectations.

### Baseline assessment

Baseline assessment will be undertaken in all participants who meet eligibility criteria and who consent to be involved in the study.

#### Baseline data

i. Allocation (control or intervention)
ii. Demographics (including ethnicity)
iii. Drivers for readmission based on outcomes of a semi-structured interview at admission
iv. Smoking and alcohol consumption
v. Co-morbidities (clinician diagnosed coronary heart disease, heart failure, rheumatic heart disease, diabetes mellitus, COPD/bronchiectasis, sleep related disorders (including obstructive sleep apnoea), and musculoskeletal disorders (type and areas/joints affected).
vi. Assessment of premorbid function status using the Resource Utilization Groups–Activities of Daily Living (RUG-ADL) [31, 32] and Australian Modified Karnofsky Performance Scale (AKPS) [33].
vii. Anthropometry and objective physical activity assessment including:
  - Weight, height and BMI
  - Sit-Rise Test (SRT) and six minute walk test (6MWT) and as a potential method for predicting patient survival and an objective, standardised and reproducible measure of exercise tolerance respectively [34, 35].

### Outcome measures

Determination of endpoints will occur at 12 months from enrolment and will be based on the CONSORT Statement [30] including an intention-to-treat analysis. Determination of endpoints will take account of censuring relating to participant survival or being lost to follow-up and will be blinded and subject to secondary panel review for adjudication where doubt exists regarding interpretation of end point definitions.

#### Primary endpoint

i. Number of all cause hospital admissions (/months follow-up) adjusting for survival and loss to follow-up

#### Secondary endpoints

i. Rate of associated all-cause hospital inpatient days (/months follow-up)
ii. Overall rate of emergency department attendances (/months follow-up)
iii. Days alive and out-of-hospital (including linkage with NT Government Death notifications) (/months of follow-up) according to actual versus maximal possible event-free days of survival
iv. Number of ICU/HDU admissions and bed days (/months of follow-up)
v. Time to first primary health care review following hospital discharge (days or mean days if more than one admission)
vi. Health care costs (see Health Economic Analyses below)

#### Accuracy of prognostic assessments

Any patient who is excluded based on a clinician determined expected prognosis less than 12 months will be followed up 12 months after initial assessment to determine if they are still alive. This will provide insight regarding the accuracy of clinician-based prognostic assessments for patients frequently admitted to hospital.

### Sample size

Sample size analysis is based on data derived from Alice Springs Hospital admission data during 2013 regarding patients suitable for inclusion in this study. Alice Springs Hospital data indicates more than 300 individual patients were admitted to Alice Springs hospital over the preceding 12 months who would meet inclusion criteria for this study. Overall these patients had a mean number of admissions of eight per year (standard deviation four). Based on an alpha of 0.05 and beta of 0.1 (power of 90%) and a clinically significant reduction in the number of readmissions of 25% the calculated sample size (control and intervention arms) will be 210 with 70 in the intervention arm and 140 in the control arm (ratio 1:2) [24].

### Recruitment strategies and consent

Participants will be actively recruited through Alice Springs Hospital over a 24 month period. A list of new admissions to any of the adult general medical teams will be assessed daily for patients who meet admission criteria. Twelve month prognosis will be specifically assessed by asking the treating specialist physician whether they anticipate a potential participant will be alive in 12 months. If the treating clinician believes the patient will be alive, or is unsure whether this is likely, they will be deemed as potentially eligible. Such patients will be assessed against inclusion/exclusion criteria and, if eligible, will be approached by staff to invite them to take part in the study. We have ethics approval to follow-up patients deemed to have a prognosis less than 12 months at the end of the trial with no additional consent required (HREC-13-159).

The consent process will include explaining the details of the study with particular reference to the study design, the possibility participants will be allocated to a control/usual care group, the nature of the intervention and the confirmation that refusal or later withdrawal will not have implications for ongoing health care. The participant consent and information forms can be found in Additional files 1 and 2. Where appropriate the Aboriginal Health Practitioner member of the research team will facilitate these discussions to ensure comprehension and cultural security for Indigenous Australian patients. If necessary local Aboriginal language translators conversant in Central Australian languages (including Arrernte, Warlpiri, Yankunytjatjara, Pitjantjatjara, Luritja, Pintupi-Luritja, Ngaatjatjarra, Ngaanyatjarra, Alyawarra and Anmatyerre) and local English idioms will be used if standard English comprehension is limited [36]. A written English language information sheet will also be provided and consent to participate confirmed with a signed and witnessed consent form.

### Time Schedule (As per SPIRIT Guidelines)

ᅟ

### Data management

All information will initially be recorded by the research team on paper forms. Paper records will be stored under numerical code in a locked filing cabinet only accessible to the study personnel. All information collected will be kept strictly confidential. A member of the research team will transpose this information into a password protected Access database on password protected computers. Data entry will be independently checked by a second member of the research team to ensure accuracy of data entry. All case files and recorded and entered data will be randomly assessed by the project manager to ensure protocol adherence (including that relating to allocation, randomisation and consent) and that there are no data omissions or errors in transcription.

**Table:** 

	STUDY PERIOD							
	Enrolment	Allocation	Post-allocation					Close-out
TIMEPOINT**	*-t*_*1*_	0	*t*_*1*_	*t*_*3*_	*t*_*6*_	*t*_*9*_	*t*_*12*_	*t*_*12*_
ENROLMENT:								
Eligibility screen	X							
Informed consent	X							
Allocation		X						
INTERVENTIONS:								
Transitional care program			X	X	X	X	X	
ASSESSMENTS:								
*≥18yrs, history of ≥4 admissions, prognosis >12mths*	X							
*Number of readmissions, Days alive/out of hospital, ICU bed days, ED presentations, time to GP review post discharge*								X
*Six minute walk test*			X	X	X	X	X	X

### Data monitoring

A Data Safety Monitoring Committee (DSMC) has been created and will be convened in the event of a serious adverse event or protocol deviation. The DSMC will request documentation from the project team and will assess, in the case of a serious adverse event, whether this was study related. The only serious adverse event monitoring during this study will be death of a participant. Any serious adverse events will be reviewed by the DSMC and reported to the supervising ethics committee. Any protocol deviations or alterations to the study protocols, consent procedures, recruitment process or study materials require review and approval by the supervising ethics committee.

An interim analysis will be undertaken after the first 80 participants have been enrolled. This analysis will focus on incidence density of readmissions and mortality. These data will be reviewed by the DSMC. A recommendation will be provided to the investigators regarding whether to continue the trial or, in the case of concerns regarding harm or clear benefit, whether to prematurely terminate the study.

### Statistical analysis

Statistical analysis of the primary end point will be based on bivariate analysis for continuous variables for inpatient days and chi square and survival analysis for all-cause mortality. Multivariate analysis will be utilised to develop predictive equations for risk of readmission and survival utilising baseline data including functional assessment results (Resource Utilization Groups–Activities of Daily Living (RUG-ADL) [31, 32], Australian Modified Karnofsky Performance Scale (AKPS) [33] and objective [6MWT, SRT [34, 35]), measures of function, co-morbidities (including severity), demographics and anthropometry. Analysis will be based on intention to treat. If data is found to be missing, this item will not be included which will be associated with deletion of cases for the prescribed elements.

### Health economic analyses

The use of all health care resources will be measured and multiplied by the respective unit costs. In general, hospital admissions typically incur higher costs for the initial period when diagnostic tests and surgical procedures are undertaken. The later days stay are relatively lower cost with little more than hotel costs incurred. The use of National Weighted Activity Units will allow standardisation and categorisation of each readmission episode into fixed and variable costs [37]. Fixed costs are those that all surviving patients incur irrespective of their length of stay, whilst variable costs are a per diem cost and are dependent on the length of stay. The total costs will be calculated as the variable costs multiplied by the length of stay plus the fixed cost. Fixed costs will include components for the emergency department, pathology, imaging, allied health professional input, pharmacy, critical care, prostheses, operating room procedures and specialist procedure suites. Per diem costs will include those for medical staff, nursing staff, non-clinical staff, staff on-costs, supplies, hotel costs (e.g. meals), and depreciation. Length of stay in intensive care and critical care units (ICU/CCU), as well overall length of stay will be ascertained. Accordingly, costs will be calculated using fixed costs plus the mean per diem cost for ICU/CCU plus the per diem cost for a general ward multiplied by the lengths of stay in each facility. Costs for providing the intervention will be calculated based on time spent by all team members with each patient. Costs for use of primary care services will be based on number of visits multiplied by a standard cost for a GP visit at the time [38].

### Ethics

The trial has ethical approval from the Central Australian Human Research Ethics Committee (HREC-13-159) and has been registered with the Australian New Zealand Clinical Trials Registry (ACTRN12615000808549). It is supported by Alice Springs Hospital and major community-based stakeholders. The trial will be conducted in compliance with the study protocol, the principles of good clinical practice [39], the National Statement on Ethical Conduct in Human Research [40], The National Health and Medical Research Council’s Values and Ethics: Guidelines for Ethical Conduct in Aboriginal and Torres Strait Islander Health Research [41]. Baker IDI indemnifies for the trial and all participants.

## Discussion

Emergency waiting times, ambulance ‘ramping’ and hospital waiting lists are all related to a lack of inpatient hospital beds. Initiatives that reduce hospital admissions can lessen emergency department overcrowding, improve patient outcomes and leverage additional value from existing hospital facilities at small cost and without the need for additional hospital beds. While this applies to all Australians the need is even greater for Indigenous Australian patients who face a burden of complex chronic disease as well as language and cultural barriers when engaging with the mainstream health system.

Comprehensive discharge planning and transitional care have been found to be effective in reducing recurrent admissions to hospital and facilitate re-engagement with primary care after discharge from hospital. Whether such findings can be transferred and adapted to a regional and remote setting with a culturally diverse patient population that has complex health care needs and is subject to substantial socioeconomic disadvantage remains to be seen.

This will be the first study to evaluate a tailored multidimensional transitional care intervention to prevent readmission in Indigenous and non-Indigenous Australian residents of remote Australia who are frequently admitted to hospital. If demonstrated to be effective it will have implications for the care and management of Indigenous people throughout regional and remote Australia. It will further be a practical demonstration and provide valuable insights into how local health care providers, including community-controlled health organisations, can provide leadership and coordination of existing clinical services and facilitate service improvements and health care savings in this setting.

## Additional files

Additional file 1: Participant consent form. Trial consent form. (DOCX 267 kb)
Additional file 2: Participant information form. Trial information form provided to patients during consenting process. (DOCX 250 kb)

## Abbreviations

6MWT: Six minute walk test
AKPS: Australian modified Karnofsky performance scale
ASHRAPP: Alice Springs Hospital Readmission Prevention Project
CAHREC: Central Australia Human Research Ethics Committee
CCU: Coronary care unit
COPD: Chronic obstructive pulmonary disease
DSMC: Data safety and monitoring committee
ICU: Intensive care unit
RUG-ADL: Resource utilization groups–activities of daily living
SRT: Sit rise test
