# Supplementary material for: The Alice Springs Hospital Readmission Prevention Project (ASHRAPP): a randomised control trial
Source: BMC Health Serv Res. 2017 Feb 20;17:153. doi: 10.1186/s12913-017-2077-7 (PMC5319097; doi:10.1186/s12913-017-2077-7)
Supplement: Additional file 1: — Participant consent form. Trial consent form. (DOCX 267 kb) [file 12913_2017_2077_MOESM1_ESM.docx]

**Participant Consent Form**

I have read and understood the *Information for Participants* and had the study explained to me and I understand what is involved.

I agree to participate in the study in the ways marked below:

☐ I agree to be a participant in ASH RAPP and understand I can withdraw at any time

☐ I agree to the researchers looking at my medical records

☐ I have had the opportunity to discuss taking part in this research with a family member or friend

☐ I understand that only those people working with the research project will have access to the information

☐ I have been given a copy of the *Information for Participants* form to keep

☐ I have read and/or understood the information provided

The researcher has agreed not to reveal my identity, my community identity or personal details if information about this project is published or presented in any public forum

Participant’s Name (printed)………………………………………………………………………….

Signature Date

Declaration by researcher: I have given this participant information on the study, which in my opinion is accurate and sufficient for the participant to understand fully the nature, risk and benefits of the study, and the rights of the research participant. There has been no coercion or undue influence.

Researcher’s name (printed)………………………………………………………………………….

Signature Date

Note: All parties signing the Consent Form must date their own signature.

Baker IDI Heart and Diabetes Institute

PO BOX 1294,

Alice Springs NT 0871

Ph: (08) 8959 0111

Alice Springs Hospital

PO Box 2234

Gap Road

Alice Springs NT 0870
