# Supplementary material for: The Alice Springs Hospital Readmission Prevention Project (ASHRAPP): a randomised control trial
Source: BMC Health Serv Res. 2017 Feb 20;17:153. doi: 10.1186/s12913-017-2077-7 (PMC5319097; doi:10.1186/s12913-017-2077-7)
Supplement: Additional file 2: — Participant information form. Trial information form provided to patients during consenting process. (DOCX 250 kb) [file 12913_2017_2077_MOESM2_ESM.docx]

**INFORMATION FOR PARTICIPANTS**

Sometimes when people come to hospital they have to wait a long time because there are no empty beds. There are things we can do to stop people waiting and make the hospital work better. We know that people want to stay at home and do not want to spend a lot of time in hospital. If the hospital can make sure that patients get good care when they go home, then people will not need to come back to the hospital. We want to do a study to see if we can help make sure when people go home from hospital that they get good care and know what is going on so that they do not have to come back to hospital.

If you are interested we would like you to be part of this study. ASH RAPP will involve 210 people accessing Alice Springs Hospital. The study is being done by doctors and researchers at the Alice Springs Hospital & Baker IDI (Central Australia).

**To take part in the study:**

- You must be aged 18 and over
- You must have been admitted to Alice Springs Hospital at least 4 times in the last year

### You cannot be part of the study if you:

- have bad kidney disease
- have had a kidney or other organ transplant
- are being looked after by the palliative care service

### What will happen if you take part in the study?

If you agree to take part, you will be asked to sign the Participant Consent Form. It is important that you understand what the research is about and that you agree to all statements on this form. You will talk to the researchers about where you come from, your health and we will check your health record for any health problems. You will also be asked to show how far you can walk before going home. Some people will get extra education about their health and medicines and we will work with their local doctor and clinic to make sure they are seen within a week of going home. If people come back to hospital within the year, we will also check how they are going. You will not know which group you belong to until after you have agreed to participate in this study.

**REMEMBER YOU CAN SAY NO AT ANY TIME AND THIS WON’T CHANGE THE CARE YOU GET FROM THE HOSPITAL, YOUR CLINIC OR GENERAL PRACTITIONER**

### Follow up

The researchers will follow-up with the hospital and clinic to find out if you are well, whether you have been seen by the clinic and if you have come back to hospital.

**Confidentiality and Risks**

We will make sure no one else but the researchers can see this information by locking all forms in a cupboard or on a computer. We will also ask you to show us how far you can walk over six minutes. This might make you short of breath but is otherwise safe. Further Information

After reading or being explained this information, please ask the researchers if there is anything you do not understand. If you have more questions you can contact **Dr Gabby Diplock** or **Chris Perry** on 8951 7777

**Ethics Approval**

This study has been approved by the Central Australian Human Research Ethics Committee.

Any person with concerns or complaints about the conduct of this study should contact:

**CAHREC Secretary**

PO Box 4066, Alice Springs, NT 0871

OR Cnr. Skinner & Simpson Sts
Alice Springs NT 0870

Ph: 08 8951 4746
E: [cahrec@flinders.edu.au](mailto:cahrec@flinders.edu.au)
